# Crucial Role for CD69 in the Pathogenesis of Dextran Sulphate Sodium-Induced Colitis

**DOI:** 10.1371/journal.pone.0065494

**Published:** 2013-06-13

**Authors:** Akihiro Hasegawa, Chiaki Iwamura, Masayuki Kitajima, Kahoko Hashimoto, Ken-ichiro Otsuyama, Hidetaka Ogino, Toshinori Nakayama, Mutsunori Shirai

**Affiliations:** 1 Department of Microbiology and Immunology, Yamaguchi University Graduate School of Medicine, Ube, Yamaguchi, Japan; 2 Department of Immunology, Graduate School of Medicine, Chiba University, Chiba, Chiba, Japan; 3 Department of Life and Environmental Sciences and High Technology Research Center, Chiba Institute of Technology, Narashino, Chiba, Japan; 4 JST, CREST, Chiba, Chiba, Japan; Charité-University Medicine Berlin, Germany

## Abstract

CD69 is a membrane molecule transiently expressed on activated lymphocytes, and its selective expression in inflammatory infiltrates suggests that it plays a role in the pathogenesis of inflammatory diseases. In this study, we used CD69-deficient (CD69 KO) mice to assess the role of CD69 in the pathogenesis of dextran sulphate sodium (DSS)-induced acute and chronic colitis. The severity of colitis was assessed by the survival rate, clinical signs, colon length, histological examination and the expression of cytokines and chemokines in the large intestines. Both acute and chronic colitis were attenuated in the CD69 KO mice, as reflected by the lower lethality, weight loss, clinical signs, and improved histological findings. CD69^+^ cells infiltrated extensively into the inflamed mucosa of the colon in WT mice after DSS treatment. Experiments with the transfer of WT CD4 T cells into CD69 KO mice restored the induction of colitis. The administration of an anti-CD69 antibody also inhibited the induction of the DSS-induced colitis. These results indicate that CD69 expressed on CD4 T cells plays an important role in the pathogenesis of DSS-induced acute and chronic colitis, and that CD69 could be a possible therapeutic target for colitis.

## Introduction

Human inflammatory bowel diseases (IBDs), such as Crohn’s disease (CD) and ulcerative colitis (UC), are characterized by chronic inflammation of the intestinal tract. The pathogenesis of IBD is related to an inappropriate and exaggerated mucosal immune responses to constituents of the intestinal flora [1]–[5]. The inflamed IBD tissue is heavily populated by inflammatory cells, including lymphocytes, plasma cells, neutrophils and macrophages [6]. Dysregulated CD4 T cells involved in adaptive immunity have also been postulated to play an important role in the pathogenesis of IBD [7]–[10]. A dysregulated T cell response leads to alterations in the mucosal cytokine expression. The patients display an impaired cytokine profile, with high local production of inflammatory cytokines including IL-1β, IL-6, IFN-γ and TNF-α [11], [12].

Dextran sulphate sodium (DSS)-induced colitis in mice has been used as a model of colitis resembling human UC. Mice that are exposed to DSS in their drinking water develop inflammation of the colon and exhibit symptoms such as diarrhea, rectal bleeding, and weight loss. DSS-induced acute colitis has been reported to be a T cell-independent model [13]. However, in chronic colitis induced by multiple cycles or in the recovery phase of DSS, adaptive immunity plays an important role in the disease process [14]–[16].

Chemokines and their receptors are considered to be important factors in the pathogenesis of IBD. Several chemokines and their receptors, including CCL2, CCL3, CCL4, CCL5, CCL17, CCL22, CXCL8, CXCL10, CCR2 and CCR5 have been documented to be up-regulated in IBD tissue [17]–[24]. CCL2 is a potent chemoattractant and an activator of monocytes [25]. CCL3, CCL4 and CCL5 recruit memory and activated CD4 and CD8 T cells [26]. Intestinal epithelial cells can rapidly produce CCL2 and CCL5 upon exposure to inflammatory mediators [27], [28]. CCR2 and CCR5 are involved in both monocyte- and macrophage-mediated immune responses, and in the regulation of T cell migration and activation. Mice deficient in CCR2 or CCR5 are protected from DSS-induced colitis [29].

CD69 is a type II membrane protein expressed as a homodimer of heavily glycosylated subunits [30]. It is known as an early activation marker antigen of lymphocytes [31], [32], and its expression is upregulated on T cells in the inflamed mucosa [33]–[35]. CD69 is also involved in the regulation of T cell egress from the thymus [36], [37] and secondary lymphoid organs [38]. We and other groups have reported a role for CD69 in the regulation of arthritis [39], [40], asthma [41], [42], myocarditis [43] and tumor immunity [44], [45]. More recently, Radulovic et al. have reported a role for CD69 in the development of colitis using a CD45RB^high^ CD4 T cell adaptive transfer model [46]. The transfer of CD69-deficient CD45RB^high^ CD4 T cells into RAG-deficient hosts induced accelerated colitis. CD69-deficient CD4 T cells showed reduced potential to differentiate into FoxP3^+^ regulatory T cells *in vivo* and *in vitro*.

We herein investigated the role of CD69 using a DSS-induced acute and chronic colitis model. In CD69-deficient (CD69 KO) mice, both acute and chronic colitis were attenuated. Cell transfer of wild-type (WT), but not CD69-deficient CD4 T cells, restored the induction of acute colitis in CD69 KO mice, indicating a critical role of CD69 expression in CD4 T cells. The infiltration of FoxP3^+^ cells was similar in the colons of DSS-treated CD69 KO mice compared with that of DSS-treated WT mice. On the other hand, the IL-10 expression was significantly increased in the colons of DSS-treated CD69 KO mice. These results suggest that the function of CD69 in the inflammatory response is complicated, but that it may represent a potential therapeutic target for Crohn’s disease or other forms of IBD.

## Materials and Methods

### Ethics Statement

All animal studies were carried out in accordance with the recommendations in the Guide for the Care and Use of Laboratory Animals of the National Institutes of Health. The protocol was approved by the Committee on the Ethics of Animal Experiments of Yamaguchi University (Permit Number: 10–008).

### Animal Studies

CD69-deficient (CD69 KO) mice [39] were backcrossed with BALB/c mice 15 times. The BALB/c were purchased from Charles River Laboratories (Tokyo, Japan). Mice were maintained under specific-pathogen-free conditions. All animal care was in accordance with the guidelines of Yamaguchi University.

### Induction and General Assessment of Colitis

Acute colitis was induced in 7 to 8-week-old mice by adding 4% (w/v) DSS (MW 36,000–50,000; MP Biomedicals, Solon, Ohio, USA) to drinking water that was filter-purified (Millipore Corp., Billerica, Massachusetts, USA) for 7 days. From day 7 onwards, animals received normal drinking water. To induce chronic colitis, mice were administered 2% DSS on days 0–5, 10–15, and 20–25. The DSS consumption, body weight, stool consistency and fecal blood loss were recorded daily. A disease activity index (DAI) [47] was calculated as described in Table S1. On day 8, 20 or 30, mice were sacrificed. After measuring the colon length, one half of the colon was fixed in 10% (vol./vol.) formalin, paraffin embedded and stained for a histological examination. The other half was frozen in liquid nitrogen and used for cytokine measurements and RNA extraction. For the anti-CD69 antibody treatment, mice were intraperitoneally injected with an anti-CD69 mAb (H1.2F3, 500 µg/mouse) on day 0. The data presented are representative of at least three individual experiments.

### Colon Histology and Immunohistochemistry

Mice were sacrificed by CO_2_ asphyxiation on day 8, and the colons were fixed with 10% (vol./vol.) formalin in PBS and embedded in paraffin. The samples were sectioned and stained with hematoxylin and eosin to examine the pathological changes under a light microscope. Colon specimens were embedded in Tissue-Tek OCT compound, frozen in liquid nitrogen, and cut by a cryostat into 6 µm thick sections. The endogenous peroxidase activity, as well as nonspecific protein binding, was sequentially blocked using 0.6% hydrogen peroxide and the Biotin-Blocking System reagent (DAKO, Glostrup, Denmark), respectively. The sections were incubated with a rabbit anti-CD3 antibody (DAKO), hamster anti-CD69 mAb (AbD, Oxford, Uk) or a rat anti-FoxP3 mAb (eBioscience, San Diego, California, USA) at 10 µg/ml overnight at 4°C and were then washed in TBST. Bound Ab was detected by sequential incubation with biotinylated rabbit anti-hamster IgG and streptavidin-HRP, followed by 3,3-diaminobenzidine (DAKO). Slides were then washed in water and counterstained with hematoxylin.

### Adoptive Transfer of CD4 T Cells and Neutrophils

Splenic CD4^+^ T cells from wild-type (WT) BALB/c mice were purified using a CD4^+^ T cell isolation kit (Miltenyi Biotec, Bergisch Glad-bach, Germany) and an Auto-MACS sorter (Miltenyi Biotec), yielding a purity of >98%. These cells were administered intravenously through the tail vain to CD69-KO mice (3×10^7^ cells/mouse) on day –1. For neutrophil preparation, wild-type BALB/c mice were injected intraperitoneally with 2 ml of 4% thioglycolate (Merck, Darmstadt, Germany), and peritoneal neutrophils were recovered 4 h later by collecting the peritoneal lavage with 5 ml of saline [48]. Neutrophils in the peritoneal lavage were stained with biotin-conjugated Gr-1 and streptavidin microbeads, then purified using an Auto-MACS sorter, yielding a purity of >90%. Fifteen million neutrophils were injected intravenously into CD69 KO mice on days 0 and 2.

### Cytokine and Chemokine Expression in the Colon Determined by Quantitative RT-PCR

The total colonic RNA was extracted using Trizol (Invitrogen, Carlsbad, California, USA). The RNA concentration was determined spectrophotometrically, and the sample quality was assessed after agarose electrophoresis. The cDNA synthesis and Quantitative RT-PCR were performed as described previously [49]. The primers and Taq Man probes used for the detection of IL-1β, IL-6, IL-10, CCR2, CCR3, CCL2, CCL4, CCL5 and HPRT were purchased from Applied Biosystems. The expression was normalized to the HPRT signal.

### Isolation of Lamina Propria (LP) Cells

The colons were removed, washed in HBSS containing 5% FCS, cut into small pieces, and incubated with HBSS containing 5% FCS and 1 mM DTT for 40 min with gentle agitation to remove epithelial cells. Tissue specimens were then incubated while being shaken in HBSS containing 5% FCS with 1.5 mg/ml collagenase IV (SIGMA-Aldrich, St. Louis, Missouri, USA) at 37°C for 40 min. The supernatant was centrifuged and the pellet was washed with RPMI 1640. LP cells were isolated by Percoll (GE Healthcare, Uppsala, Sweden) density gradient centrifugation (800×g for 25 min) and collected at the interface. If necessary, CD4^+^ cells were isolated by magnetic negative selection with the CD4 MACS system (Miltenyi Bictec).

### Immunofluorescent Staining and Flow Cytometric Analysis

In general, one million cells were incubated on ice for 30 min with the appropriate staining reagents, according to a standard method [50]. The intracellular staining of IL-10 was performed as described previously [51]. A PE-conjugated anti-IL-10 mAb (JES5-16E3, BD Biosciences, San Jose, California, USA), FITC-conjugated anti-Gr-1 mAb (RB6-8C5, BD Biosciences), APC-conjugated anti-CD4 mAb (RM4-5, BD Biosciences), anti-CD8a mAb (53-6.7, BD Biosciences), anti-CD11b mAb (M1/70, eBioscience) and anti-CD11c mAb (N418, BioLegend, San Diego, California, USA) were used for detection of the target proteins.

### Proliferation Assay

Splenic CD4^+^ T cells (2×10^5^) prepared by the AutoMACS sorter were stimulated in 200 µl cultures for 40 h with immobilized anti-TCRβ mAb (H57-597, BD Biosciences), PMA (50 ng/ml) and ionomycin (500 nM). [^3^H]Thymidine (37 kBq/well) was added to the stimulation culture for the last 16 h, and the incorporated radioactivity was measured on a beta plate [51].

### Analysis of the Efficiency of Cell Division

CD4^+^ T cells isolated from lamina propria were labeled with CFSE (Invitrogen) as described previously [52]. The cells were cultured in complete medium in the presence of Con A (2 µg/ml) (WAKO, Osaka, Japan) for 48 h, and then were analyzed on a FACSCalibur instrument (BD Biosciences).

### Statistical Analysis

The disease activity indices were statistically analyzed using the Mann-Whitney U test. Differences in parametric data were evaluated by Student’s *t*-test. Differences of p<0.05 were considered to be statistically significant. The variance of the groups was tested for equality by the *F* test prior to the *t*-test.

## Results

### Improvement of DSS-induced Acute Colitis in CD69 KO Mice

**Figure 1:**
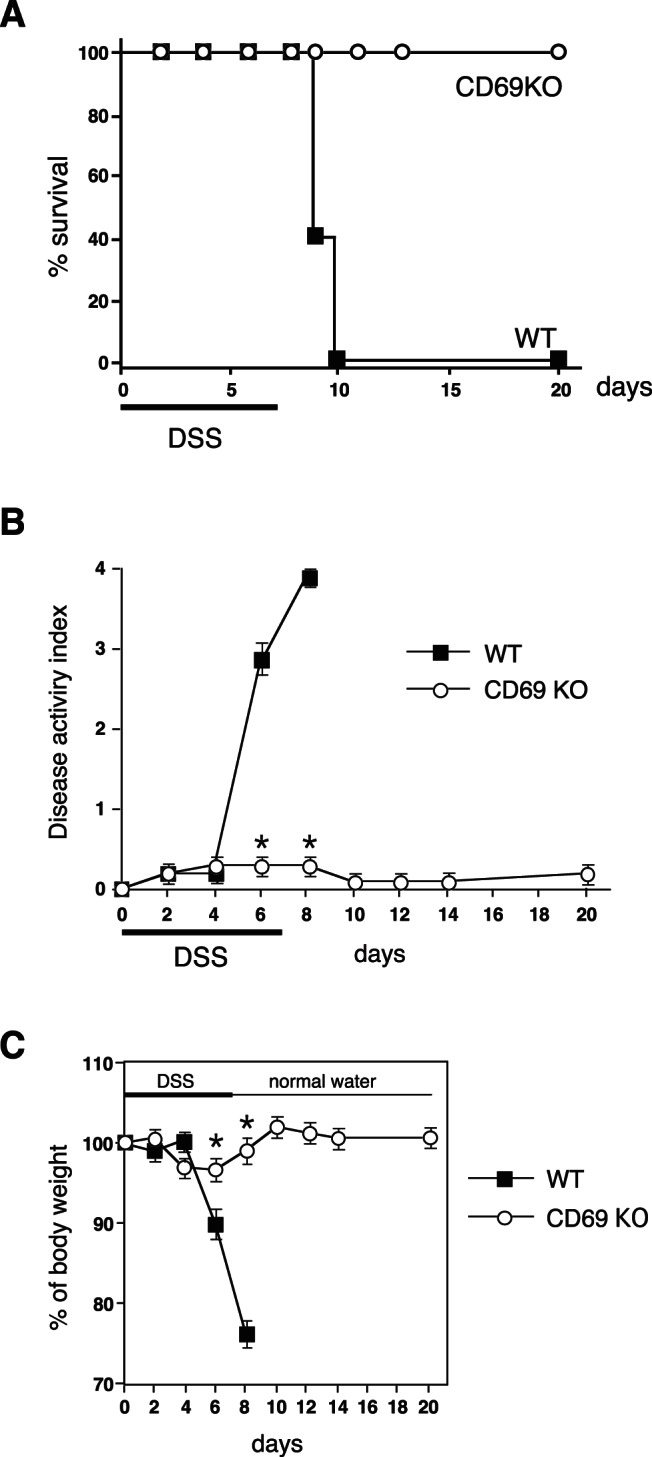
Inhibition of dextran sulphate sodium (DSS)-induced acute colitis in CD69-deficient (CD69 KO) mice. Acute colitis was induced by giving animals 4% DSS in drinking water for 7 days, followed by normal drinking water. (A) The survival rates of CD69 KO and wild-type (WT) mice after the initiation of during DSS-induced acute colitis. The survival was recorded daily (n = 10 per group). (B, C) Changes in the disease activity index (B) and body weight (%) (C) over the course of DSS treatment in CD69 KO and WT mice. The data are presented as the means (SD) (n = 10 per group). *p<0.01. Three independent experiments were performed with similar results.

In order to evaluate the role of CD69 in the development of experimental colitis, acute colitis was induced in CD69 KO and control wild-type (WT) mice by adding 4% DSS to their drinking water. The survival rates were significantly higher in CD69 KO mice compared with those in control WT mice after DSS administration (Fig. 1A). CD69 KO mice showed significant protection against DSS-induced acute colitis, as indicated by the weight loss and clinical scores for weight loss, bleeding, and diarrhea (Fig. 1B and 1C). On day 8, the DSS-treated WT mice lost more than 20% of their body weight (maintaining 76.5±2.8% of their original weight, Fig. 1C). In contrast, the DSS-treated CD69 KO mice remained active, and their body weight was 99.0±2.0% of their original weight on day 8.

**Figure 2:**
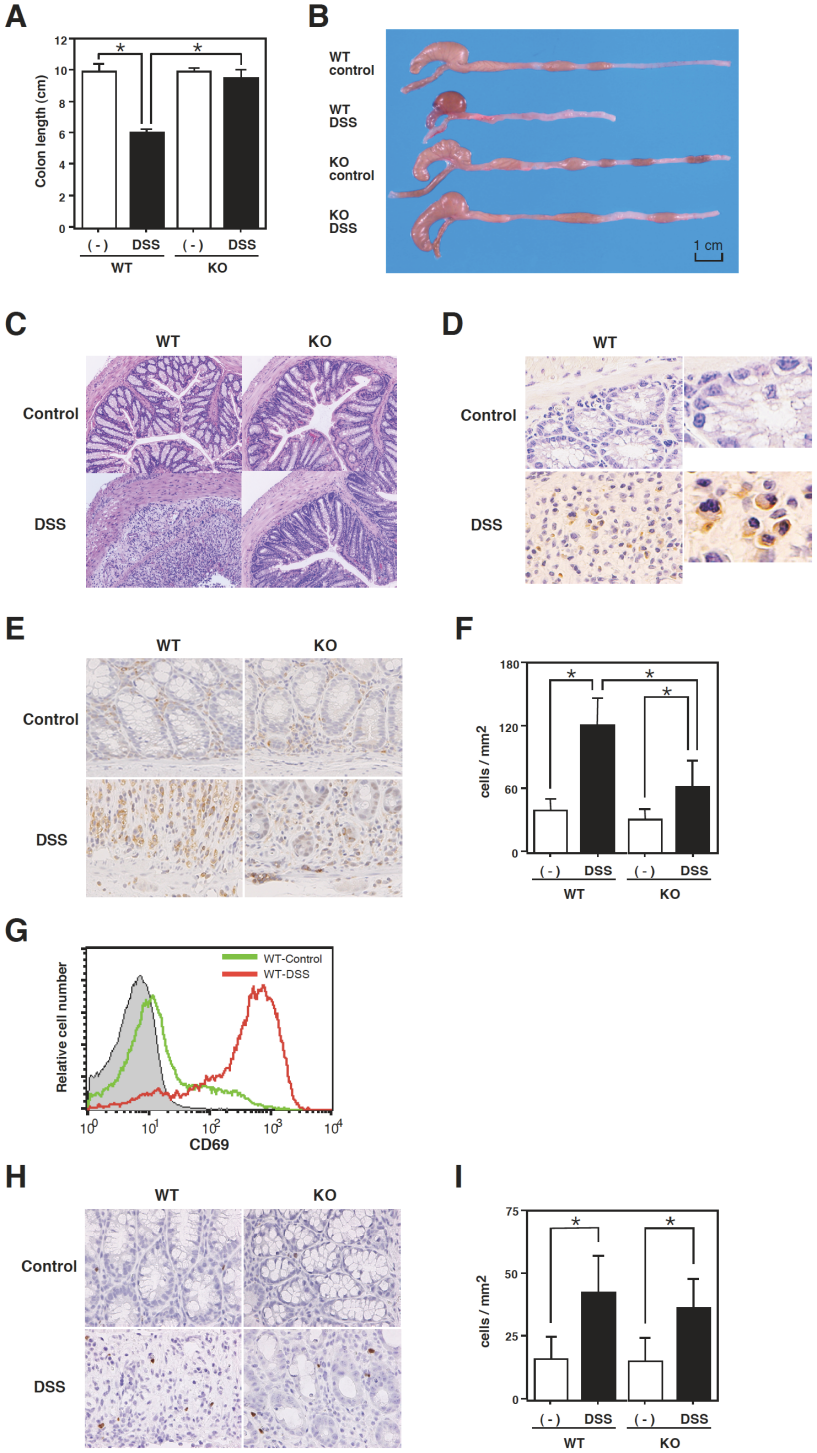
DSS-induced colocecal damage was reduced in CD69 KO mice. (A) The colon length. On day 8, the colon length of DSS-exposed wild-type (WT) and CD69 KO mice and control mice was measured. The data are presented as the means (SEM) (n = 12 per group). *p<0.01. (B) A representative photograph showing the gross appearance of the colon from each group is shown. (C) Histological sections of inflamed colons. Colons were taken on day 8 from WT and CD69 KO mice that received DSS in drinking water. Sections were prepared and stained with hematoxylin and eosin (original magnification 40×). (D) Immunohistochemical staining of CD69-positive cells in the colonic tissues of DSS-treated WT mice (original magnification 300× and 600×). (E, H) Immunohistochemical staining of CD3-positive cells (E) and FoxP3-positive cells (H) in the colonic tissues of DSS-treated WT and CD69 KO mice (original magnification 200×). (F, I) Summary of the accumulation of CD3-positive cells (F) and FoxP3-positive cells (I). Data are from 20 fields from 5 mice. *p<0.01. (G) The expression of CD69 on electronically gated lamina propria CD4 T cells from control (green line) and DSS-treated (red line) WT mice. Background staining is shown as hatched areas.

The colon length progressively shortened and luminal bleeding was observed in WT mice on day 8 after DSS administration (Fig. 2A and 2B). The severity of changes in the gross appearance and in luminal bleeding was significantly lower and the colon length was maintained in the CD69 KO mice (Fig. 2A and 2B). During the histological examination, crypt damage, ulceration, and infiltration of inflammatory cells were observed in the colons of DSS-treated WT mice (Fig. 2C). On the other hand, the histological analysis of colons from DSS-treated CD69 KO mice showed greatly reduced numbers of infiltrating cells, a lower degree of mucosal injury, and less edema. These results indicate that the DSS-induced acute colitis was attenuated in CD69 KO mice.

### Expression of CD69 Molecules on Infiltrating Cells in the Colon

Next, we examined the expression of CD69 by the infiltrating cells in the colon of WT mice treated with DSS. Immunohistochemical staining was performed on the colons from DSS-treated WT mice. As shown in Fig. 2D, significant expression of CD69 was detected on the infiltrating cells in the colons of DSS-treated WT mice. However no significant expression of CD69 was observed in the colons of untreated WT mice (Fig. 2D).

### Infiltration of CD69-deficient T Cells into the Colon

To evaluate the ability of CD69-deficient T cells to infiltrate into the inflamed colon tissue, we performed immunohistochemical staining of colonic cross sections from DSS-treated WT and CD69 KO mice. As can be seen in Fig. 2E, substantial numbers of CD3^+^ cells had infiltrated into the colons of DSS-treated WT mice on day 8. The infiltration of CD3^+^ cells was significantly reduced in the colons of CD69 KO mice (Fig. 2E and 2F). These results indicate that the infiltration of CD69-deficient CD3^+^ cells in the inflamed colon was reduced in comparison to that of WT CD3^+^ cells.

We next assessed the cell surface expression of CD69 on the infiltrating CD4 T cells by flowcytometry. As shown in Fig. 2G, the cell surface expression of CD69 on the lamina propria CD4 T cells was upregulated in DSS-treated WT mice.

We also performed immunohistochemical staining for FoxP3 in the colonic cross sections from DSS-treated WT and CD69 KO mice. As shown in Fig. 2H, FoxP3^+^ cells had infiltrated into the colons of DSS-treated WT mice on day 8. The infiltration of FoxP3^+^ cells was similar in the colons of DSS-treated CD69 KO mice compared with that of DSS-treated WT mice (Fig. 2H and 2I). These results indicate that the impaired infiltration of CD69-deficient CD3^+^ cells in the inflamed colon was not due to the increased infiltration of FoxP3^+^ regulatory T cells in the inflamed colon.

### Cytokine, Chemokine and Receptor Expression in the Colon

**Figure 3:**
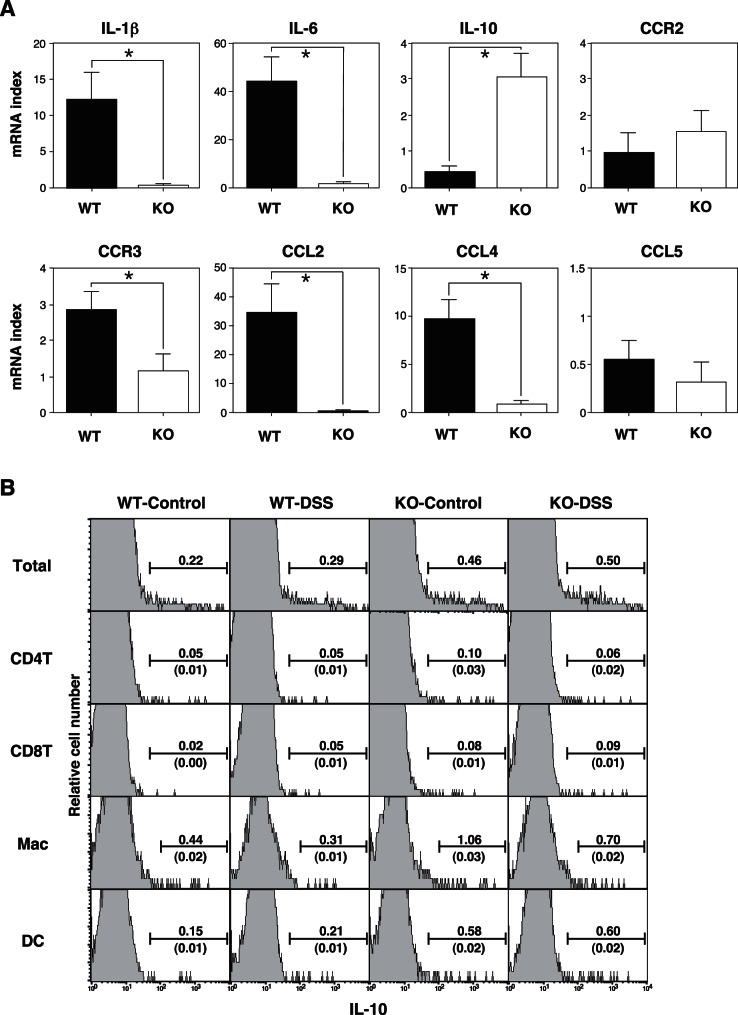
The expression of cytokines, chemokines and their receptors in the colons of DSS-treated mice. (A) The expression of cytokines, chemokines and their receptor mRNA in the colons of DSS-treated mice. Mice were sacrificed on day 8 and the colon tissues were harvested. Whole colonic RNA was isolated, reverse-transcribed into cDNA, and the expression levels of IL-1β, IL-6, IL-10, CCR2, CCR3, CCL2, CCL4 and CCL5 were determined by real-time quantitative PCR. Data are expressed as the ratios of the target mRNA levels to the HPRT mRNA level (n = 5 per group). *p<0.05. Data are presented as the means (SD). The data are representative of three independent experiments. (B) The intracellular staining profiles of IL-10 in the lamina propria CD4 T cells (CD4^+^), CD8 T cells (CD8^+^), macrophages (CD11b^+^, Gr-1^int^) and dendritic cells (CD11c^+^) are shown as the percentages of cells in each area. The ratios of IL-10^+^ cells/total cells of each cell subset are shown over the area bar. The ratios of IL-10^+^ cells in each subset/total cells of all subsets are shown under the area bar. The results are representative of three independent experiments.

DSS treatment strongly induces several inflammatory cytokines and chemokines, such as CCL2, CCL3 and CCL5. In order to examine the pattern of cytokine and chemokine expression in the DSS-treated animals, we also performed reverse transcription (RT) PCR using RNA derived from whole colonic tissue. There were no significant increases in the mRNA expression of any of these molecules in either the untreated WT or CD69 KO mice (data not shown). However, DSS treatment caused strong induction of IL-1β, IL-6, CCL2, CCL4 and CCL5 mRNA in DSS-treated WT mice in comparison with untreated mice (data not shown). The expression of IL-1β, IL-6, CCL2, and CCL4 was decreased in the colons of DSS-treated CD69 KO mice in comparison to the DSS-treated WT mice (Fig. 3A). The expression of CCR3 was partially inhibited in the colons of DSS-treated CD69 KO mice. In contrast, the expression levels of CCR2 and CCL5 were similar in the colons of DSS-treated CD69 KO mice and DSS-treated WT mice. The IL-10 expression was significantly increased in the colons of DSS-treated CD69 KO mice in comparison to DSS-treated WT mice. These results suggest that CD69 KO mice are protected from severe colitis because of the reduced induction of proinflammatory cytokines and chemokines, such as IL-1β, IL-6, CCL2 and CCL4. In addition, the higher levels of IL-10 in the colon may be involved in the protection of CD69 KO mice from severe colitis.

In order to identify the major sources of IL-10 in the colon, we performed intracellular IL-10 staining of the lamina propria cells in the colon from DSS-treated mice. The percentages of total IL-10^+^ cells in CD69 KO mice were higher than those in WT mice even in the untreated mice (Fig. 3B). The number of IL-10^+^ cells increased in the DSS-treated mice in comparison to untreated WT and CD69 KO mice. We also performed intracellular staining of CD4 T cells, CD8 T cells, macrophages and dendritic cells. The percentages of IL-10^+^ cells in all subsets in CD69 KO mice were higher than those in WT mice. However, the number of IL-10^+^ cells among these four cell subsets was less than 20% of the total IL-10^+^ cells in CD69 KO mice. Thus, the major source of IL-10 in the colon of CD69 KO mice seems to be a cell subset other than CD4 T cells, CD8 T cells, macrophages and dendritic cells.

### Requirement of CD69 on CD4 T Cells for the Induction of DSS-induced Colitis

**Figure 4:**
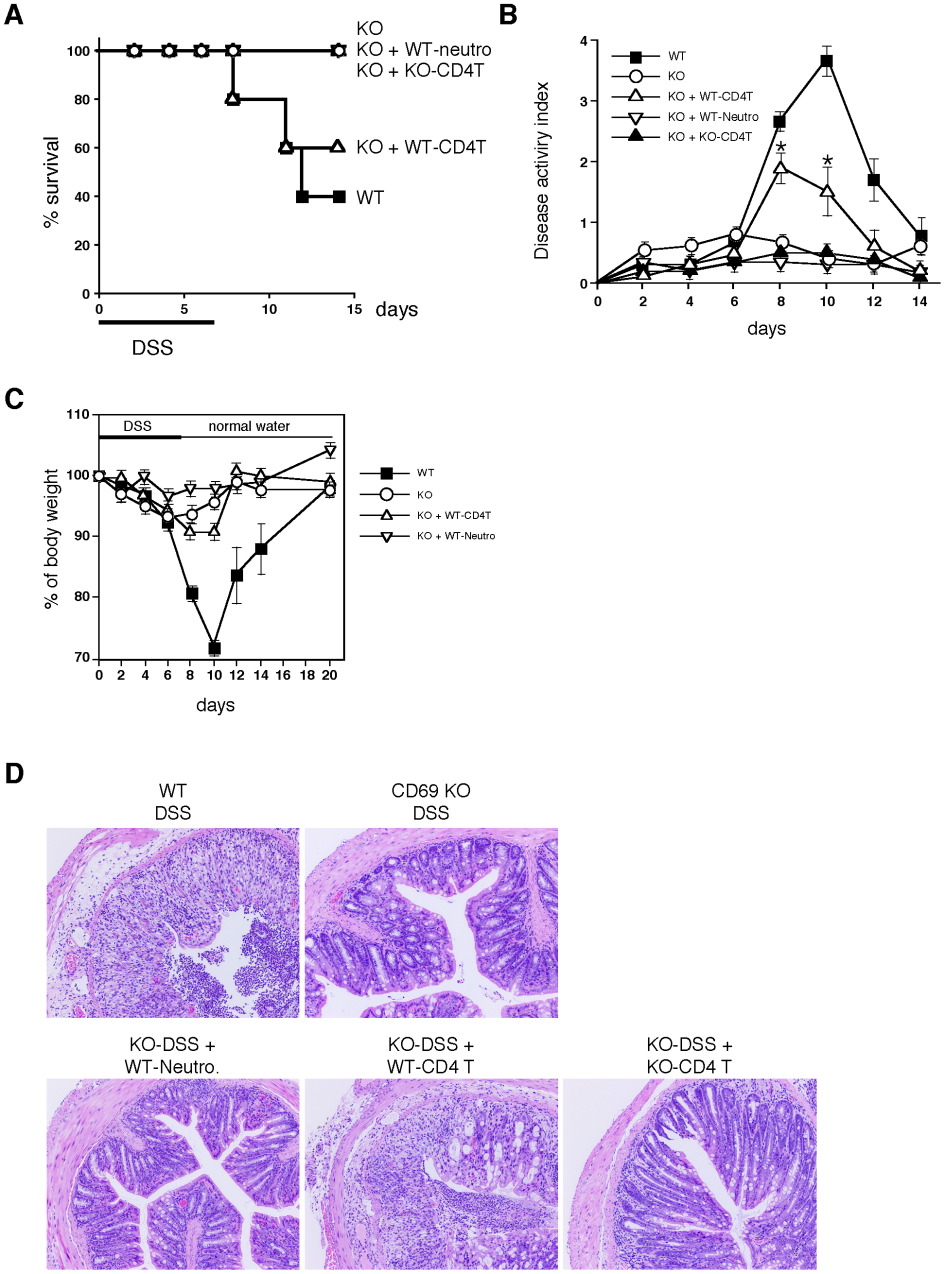
DSS-induced colitis was restored by adoptive transfer of wild-type (WT) CD4 T cells into CD69 KO mice. (A) The survival rates of WT, CD69 KO mice without cell transfer, and CD69-KO mice that received WT CD4 T cells (WT-CD4T), CD69 KO CD4 T cells (KO-CD4T), or WT neutrophils (WT-Neutro) during DSS treatment. Survival was recorded daily (n = 5 per group). (B, C) Changes in the disease activity index (B) and body weight (%) (C) over the course of DSS treatment. The data are presented as the means (SD) (n = 5 per group). *p<0.05. (D) Histological sections of inflamed colons. Colons were taken on day 8 from mice receiving DSS in their drinking water. Sections were fixed and stained with hematoxylin and eosin (original magnification 40×). Two independent experiments were performed with similar results.

To investigate the cellular basis underlying the requirement of CD69 in the pathogenesis of DSS-induced acute colitis, we performed cell transfer experiments in which splenic CD4 T cells from WT and CD69 KO mice were adoptively transferred into CD69 KO mice. As shown in Fig. 4A, the survival rates were significantly restored by the transfer of WT CD4 T cells to the CD69 KO mice. Although the weight loss did not show any significant difference following the transfer of WT CD4 T cells to the CD69 KO mice (Fig. 4C), the clinical scores for weight loss, bleeding, and diarrhea were all worsened by the transfer of WT CD4 T cells to the CD69 KO mice (Fig. 4B).

On the other hand, the transfer of WT neutrophils or CD69 KO CD4 T cells to the CD69KO mice failed to change the survival rates or clinical scores. The histological analysis revealed that the transfer of WT CD4 T cells resulted in a substantial increase in crypt damage, ulceration, and infiltration of inflammatory cells in the colon, whereas transfer of WT neutrophils or CD69 KO CD4 T cells failed to induce the inflammatory cell infiltration (Fig. 4D). These results indicate that CD69 molecules on CD4 T cells play an important role in the induction of DSS-induced acute colitis.

With regard to the CD4 T cell function, the anti-TCRβ mAb-induced proliferative responses of splenic CD4 T cells were similar between WT and CD69 KO mice (Fig. S1A). We next examined the Con A-induced cell division of lamina propria CD4 T cells. After 48 h of culture, the cells had divided two to three times in the case of both WT and CD69 KO mouse T cells. The rate of cell division of the lamina propria CD4 T cells was indistinguishable between WT and CD69 KO mice (Fig. S1B and S1C). Similar results were obtained using splenic CD4 T cells (data not shown). These results that there are no obvious differences in the proliferations of CD4 T cells from the CD69 KO mice.

### The *in vivo* Treatment with an Anti-CD69 mAb Inhibited the Induction of DSS-induced Acute Colitis

**Figure 5:**
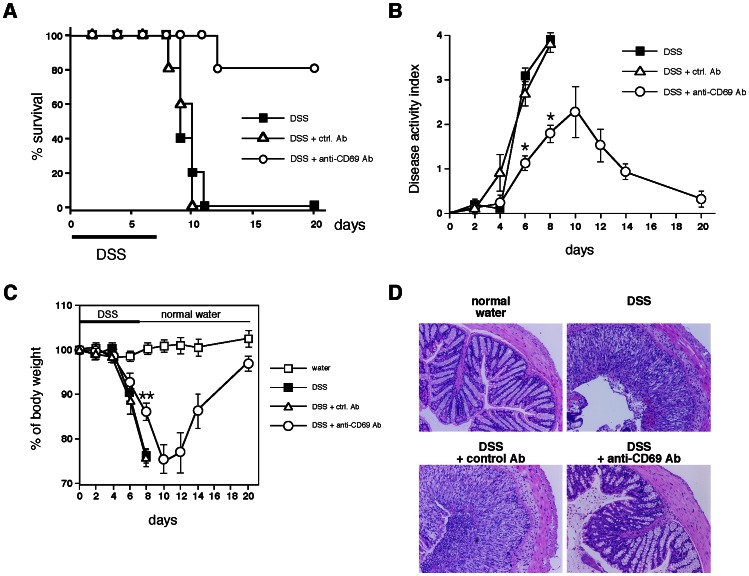
Effect of *in vivo* treatment with an anti-CD69 monoclonal antibody (mAb) on DSS-induced colitis. (A) Wild-type (WT) BALB/c mice were treated with an anti-CD69 mAb or control hamster IgG on day 0. The survival of each group during DSS-induced colitis was recorded daily (n = 5 per group). (B, C) Changes in the disease activity index (B) and body weight (%) (C) over the course of DSS treatment in WT mice treated with the anti-CD69 mAb or control hamster IgG. The data are presented as the means (SD) (n = 5 per group). *p<0.01. **p<0.05. (D) Histological sections. Colons were taken on day 8 from control and DSS-exposed WT mice treated with the anti-CD69 mAb or control hamster IgG. Sections were fixed and stained with hematoxylin and eosin (original magnification 40×). Three independent experiments were performed with similar results.

In order to explore the potential therapeutic effect of the administration of an anti-CD69 monoclonal antibody (mAb) during DSS-induced acute colitis, WT BALB/c mice were treated with 500 µg of anti-CD69 mAb or control antibody (Ab) on day 0. The survival rates were significantly increased in the anti-CD69 mAb-treated WT mice compared with control Ab-treated WT mice (Fig. 5A). Anti-CD69 mAb treated-WT mice showed significant protection against DSS-induced acute colitis, as indicated by their decreased weight loss and better clinical scores for weight loss, bleeding, and diarrhea (Fig. 5B and 5C). Furthermore, a histological analysis of the colons from anti-CD69 mAb-treated WT mice showed greatly reduced numbers of infiltrating cells, a lower degree of mucosal injury, and less edema (Fig. 5D). These results suggest that the development of DSS-induced acute colitis can be inhibited by treatment with an anti-CD69 mAb.

### Improvement of DSS-induced Chronic Colitis in CD69 KO Mice

**Figure 6:**
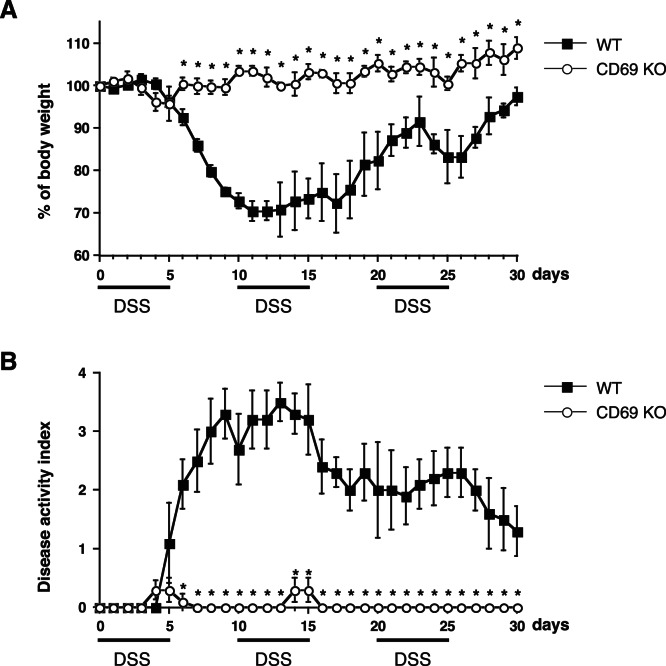
DSS-induced chronic colitis in CD69 KO mice. CD69 KO mice and wild-type (WT) mice were administered 2% DSS on days 0–5, 10–15, and 20–25. The body weight (A) and disease activity index (B) were monitored every day, and the values for body weight are expressed as the percentage of body weight on day 0. The data are presented as the means with SD (n = 5 per group). *p<0.01. Three independent experiments were performed with similar results.

To investigate the role of CD69 in the pathogenesis of DSS-induced chronic colitis, CD69 KO mice were administered 2% DSS on days 0–5, 10–15, and 20–25. As shown in Fig. 6A and 6B, DSS-induced chronic colitis was dramatically attenuated in CD69 KO mice, as indicated by the reduced weight loss and disease activity index of weight loss, bleeding, and diarrhea. The disease activity index was especially improved in the first recovery phase (days 6) of the exaggerated colitis induced by DSS. These results indicate that the DSS-induced chronic colitis is attenuated in CD69 KO mice.

## Discussion

In this study, we used CD69 KO mice to assess the role of CD69 in a DSS-induced colitis model. We have herein demonstrated that both acute and chronic colitis were attenuated in CD69 KO mice (Fig. 1 and 6), and that the CD69 expressed on CD4 T cells plays an important role in the development of colitis (Fig. 4). The attenuated colitis in CD69 KO mice appeared to be due largely to the reduced infiltration of T cells into the inflamed colon (Fig. 2E). In addition, a therapeutic effect of administering an anti-CD69 mAb was revealed (Fig. 5), indicating that CD69 could represent a new target for mAb treatment in IBD patients.

CD69 KO mice showed reduced colitis in both acute and chronic DSS-induced colitis models (Fig. 1, 2A, 2B, 2C and 6), and CD69 was highly expressed on the infiltrating CD4 T cells in DSS-treated WT mice (Fig. 2G). The infiltration of CD3^+^ cells was significantly reduced in the colons of CD69 KO mice (Fig. 2E). The DSS-induced colitis was restored by adoptive transfer of WT CD4 T cells, but not CD69-deficient CD4 T cells, to the CD69 KO mice (Fig. 4). These results indicate that CD69 molecules on CD4 T cells play an important role in the induction of DSS-induced acute colitis. The proliferative responses of CD4 T cells from the spleen and colonic lamina propria were indistinguishable between WT and CD69 KO mice (Fig. S1). Thus, the regulation of the proliferative activity of CD4 T cells may not be the mechanism by which the CD69 on CD4 T cells plays a role in inducing colitis. Although DSS-induced acute colitis has been reported to be a T cell-independent model [13], adaptive immunity plays an important role in the disease progression of chronic colitis and/or in the recovery phase following the administration of DSS [14]–[16]. Thus, CD69^+^ CD4 T cells may be involved in the disease progression of chronic colitis or the recovery from acute colitis. On the other hand, the survival rates and the clinical scores were significantly restored by the transfer of WT CD4 T cells to CD69 KO mice, but the weight loss did not show any significant differences following this restoration. These results suggest that CD69-expressing cells other than CD4 T cells may also contribute to the pathogenesis of DSS-induced colitis.

We observed that the migration of CD3^+^ cells was reduced in the colons of CD69 KO mice (Fig. 2E). CCL2 and CCL4 recruit memory and activated CD4 and CD8 T cells [26]. The expression of both CCL2 and CCL4 was dramatically inhibited in the colons of DSS-treated CD69 KO mice (Fig. 3A). Therefore, it is possible that inhibiting the induction of CCL2 and CCL4 may reduce the migration of T cells in the colon and protect CD69 KO mice from severe colitis. Although the ligand for CD69 has not been identified, another possible scenario is that a putative ligand may be induced and expressed on the inflamed colon tissues. Activated CD4 T cells expressing CD69 then may migrate into the colon tissue, and remain at the inflammatory site efficiently via the CD69/CD69 ligand interaction. CD69-deficient CD4 T cells may not be retained at the inflammatory site very efficiently because there would be no interaction between CD69 and its ligand(s).

Another potential mechanism could be that the CD69 expressed on CD4 T cells may regulate the induction and/or maintenance of inflammation in the colon tissue (Fig. 4). Interestingly, difference in the migration of FoxP3^+^ regulatory T cells in the colon was not detected in the CD69 KO mice (Fig. 2H). On the other hand, IL-10 expression was significantly increased in the colons of DSS-treated CD69 KO mice (Fig. 3). Higher expression levels of IL-10 in the colon may be involved in the protection of CD69 KO mice from severe colitis. Intracellular IL-10 staining of the lamina propria cells in the colon revealed higher percentages of IL-10^+^ cells in even untreated CD69 KO mice (Fig. 3B). IL-10 is a cytokine that is predominantly secreted by CD4 memory and effector T cells, regulatory T cells and antigen-presenting cells, such as monocytes/macrophages. The number of IL-10^+^ cells in the total population of CD4 T cells, including CD4^+^ FoxP3^+^ regulatory T cells, and CD11b^+^Gr-1^int^ macrophages was less than 20% of the total IL-10^+^ cells in CD69 KO mice. These results suggest that the major source of IL-10 in the colon of CD69 KO mice seems to be a cell subset other than these cells. Therefore, further investigations will be required to identify the major cell source of IL-10 and the molecular mechanism(s) underlying the high IL-10 expression in CD69 KO mice.

This study demonstrated the therapeutic effect of the administration of an anti-CD69 mAb (Fig. 5). The ligand for CD69 has not been identified, but it is possible that the anti-CD69 mAb may block the interaction between CD69 and putative CD69 ligands, resulting in reduced CD4 T cell migration and attenuated colitis. Another mechanism that may explain the inhibitory effect of anti-CD69 antibody treatment could be the downregulation of CD69 molecules on CD4 T cells. It is possible that CD69 may transduce some type of inhibitory signal to reduce CD4 T cell activity. A third potential mechanism could be antibody-dependent cellular cytotoxicity (ADCC) resulting from the anti-CD69 mAb, but this is unlikely, because no evidence supporting ADCC was obtained in our previous study [41].

Recently CD69 has been suggested to increase the incidence and severity of colitis in a T cell transfer colitis model [46]. In this model, colitis was induced by transplanting RAG KO mice with CD45RB^high^ CD4 T cells. Radulovic et al. demonstrated that the CD4 T cells from CD69 KO mice produced higher amounts of the proinflammatory cytokines IFN-γ, TNF-α, and IL-21, whereas the production of TGF-β1 was decreased. The transfer of CD69-deficient CD45RB^high^ CD4 T cells into RAG KO hosts induced accelerated colitis. CD69-deficient CD4 T cells showed a lower potential to become FoxP3^+^ regulatory T cells. In contrast, in our present study using a model of DSS-induced colitis, the FoxP3^+^ regulatory T cells migrated normally in the inflamed tissue (Fig. 2H), and impaired colitis was observed in both the acute and chronic models of colitis (Fig. 1, 4, and 6). It appears that the contribution of Treg cells to the pathogenesis of colitis in these two models may be different. This could be the reason why apparent contradictory results in CD69-deficient mice were obtained in these two models. CD69 may play an important role in the induction of inflammation as a result of its expression on effector T cells, and also in the inhibition of inflammation by Treg cells.

In summary, our results indicate that the CD69 expressed on CD4 T cells plays a critical role in the development of DSS-induced acute and chronic colitis via the efficient migration of activated CD4 T cells into the colon. Moreover, our mAb administration experiments revealed that CD69 could be a potential therapeutic target for inflammatory bowel disease.

## Supporting Information

Figure S1 **Functional characterization of CD69 KO mouse T cells.** (A) Splenic CD4 T cells from WT and CD69 KO mice were stimulated with immobilized anti-TCRβ mAb or PMA (50 ng/ml) plus ionomycin (500 nM). The mean [^3^H]thymidine incorporation of each group is shown with SDs. (B, C) CD4 T cells isolated from the lamina propria were labeled with CFSE and stimulated with Con A (2 µg/ml). After culturing them for 48 h, the number of cell divisions (0 to 4) was assessed by flow cytometry (B), and the percentages of the cells in the gates representing the different numbers of cell divisions are shown (C). Three independent experiments were performed and similar results were obtained each time.
(TIFF)

Table S1 **Scoring of disease activity index.** The disease activity index is the combined scores of weight loss, stool consistency and bleeding divided by three. ^a^Normal stools = well-formed pellets, loose stools = pasty and semi-formed stools which do not stick to the anus, diarrhea = liquid stools that stick to the anus.
(TIFF)
