# Supplementary figures and images for: Crucial Role for CD69 in the Pathogenesis of Dextran Sulphate Sodium-Induced Colitis
Source: PLoS One. 2013 Jun 13;8(6):e65494. doi: 10.1371/journal.pone.0065494 (PMC3681816; doi:10.1371/journal.pone.0065494)

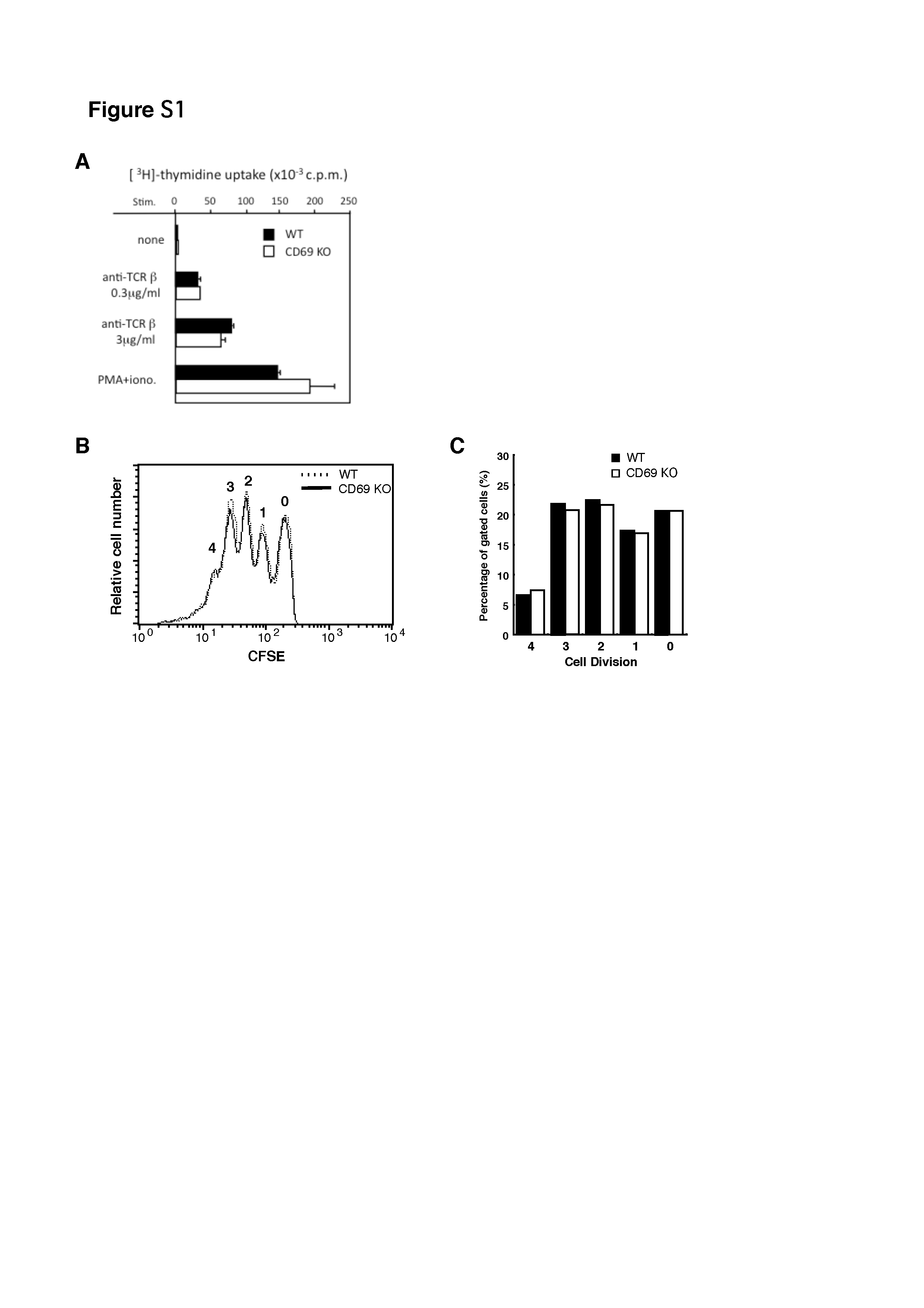

Supplement: Figure S1 — Functional characterization of CD69 KO mouse T cells. (A) Splenic CD4 T cells from WT and CD69 KO mice were stimulated with immobilized anti-TCRβ mAb or PMA (50 ng/ml) plus ionomycin (500 nM). The mean [3H]thymidine incorporation of each group is shown with SDs. (B, C) CD4 T cells isolated from the lamina propria were labeled with CFSE and stimulated with Con A (2 µg/ml). After culturing them for 48 h, the number of cell divisions (0 to 4) was assessed by flow cytometry (B), and the percentages of the cells in the gates representing the different numbers of cell divisions are shown (C). Three independent experiments were performed and similar results were obtained each time. (TIFF) [file pone.0065494.s001.tiff]
